# Imaging Spectrum of Thoracic Outlet Syndrome: A Case Series Highlighting Arterial, Venous, and Neurogenic Subtypes

**DOI:** 10.7759/cureus.108620

**Published:** 2026-05-10

**Authors:** Nishitha Utukuru, Bhargav Reddy, Rajoo Ramachandran

**Affiliations:** 1 Department of Radiology and Imaging Sciences, Sri Ramachandra Institute of Higher Education and Research, Chennai, IND

**Keywords:** cervical rib, ct venography, doppler ultrasound, subclavian vessels, thoracic outlet syndrome

## Abstract

Introduction: Thoracic outlet syndrome (TOS) encompasses a spectrum of neurovascular compression disorders involving the brachial plexus, subclavian artery, and subclavian vein. Diagnosis remains challenging due to variable clinical presentations and limited specificity of imaging findings, particularly in neurogenic TOS. This case series aims to demonstrate the multimodality imaging spectrum of TOS across arterial, venous, and neurogenic subtypes, compare imaging appearances among subtypes, and emphasise the importance of clinicoradiological correlation in diagnosis.

Methods: This retrospective case series was conducted at Sri Ramachandra Medical Health Centre, Chennai, Tamil Nadu, India, from January 2020 to March 2026. Eight consecutive patients with clinical suspicion of TOS (upper limb pain, swelling, venous engorgement, neurological symptoms, or ischaemic changes) underwent imaging evaluation. Imaging included Doppler ultrasound with provocative manoeuvres, computed tomography angiography (CTA), CT venography (CTV), and magnetic resonance imaging (MRI), tailored to clinical presentation. Imaging was assessed for osseous anomalies, site of compression across thoracic outlet compartments, vascular stenosis or occlusion, thrombosis, aneurysmal change, collateralisation, and indirect signs of brachial plexus compression.

Results: Imaging demonstrated a broad spectrum of pathology across all TOS subtypes. Arterial TOS cases showed dynamic or fixed subclavian artery compromise, ranging from focal costoclavicular stenosis detected on CTA to long-segment chronic occlusion associated with an anomalous first rib. Venous TOS cases demonstrated subclavian vein compression at the costoclavicular space, including McCleery syndrome without thrombosis, muscular compression from a hypertrophied subclavius muscle, and chronic obstruction with extensive collateral venous channels. One patient demonstrated multi-level venous obstruction involving the subclavian and cephalic veins with associated central venous thrombosis. Neurogenic TOS demonstrated thoracic outlet narrowing due to a cervical rib with displacement of adjacent neurovascular structures on MRI. Dynamic Doppler imaging was critical in demonstrating haemodynamically significant stenosis not evident at rest.

Conclusion: This case series demonstrates the broad imaging spectrum of TOS, ranging from dynamic arterial stenosis and chronic arterial occlusion to venous compression with collateralisation and indirect signs of neurogenic involvement. The cases highlight that the imaging appearance varies significantly depending on the affected structure and the site of compression within the thoracic outlet. Recognition of characteristic vascular findings such as focal costoclavicular narrowing, thrombosis, chronic occlusion, and collateral venous pathways can facilitate subtype classification and guide further management. However, imaging findings must be interpreted in conjunction with clinical symptoms, particularly in neurogenic TOS, where radiologic abnormalities may be subtle or absent.

## Introduction

Thoracic outlet syndrome (TOS) is defined as a collection of symptoms due to compression of the neurovascular bundle as it passes through the thoracic outlet, which includes the brachial plexus, subclavian artery, and subclavian vein [1]. TOSs can be classified based on the structures involved, which include neurogenic, venous, and arterial varieties, each displaying its own clinical and imaging characteristics [1]. 

The thoracic outlet represents a complex anatomical region that extends from the cervical spine down to the subcoracoid area and is divided into three spaces: the interscalene triangle, the costoclavicular space, and the retropectoralis minor space [1-3]. Compression may arise from congenital factors such as cervical ribs or fibrous bands or from acquired causes, including trauma, repetitive activity, and postural abnormalities [1,4]. 

The diagnosis of TOS remains challenging due to its non-specific clinical presentation. Although several diagnostic approaches and consensus recommendations have been proposed in the literature, universally accepted standardised diagnostic criteria remain lacking, particularly for neurogenic TOS. Neurogenic TOS represents most cases, where signs and symptoms are vague and difficult to confirm on imaging studies [5-7]. On the contrary, vascular variants are relatively rare, but they usually have specific radiological findings, such as thrombosis, stenosis, development of an aneurysm, or distal emboli [1,3]. 

The role of imaging in the assessment of patients with TOS is crucial, primarily for identifying anatomical abnormalities, assessing vascular compromise, localising the site of compression, and excluding other diseases [1]. However, it must be borne in mind that the imaging results should be correlated with the clinical picture, as some people with anatomical variations or even vascular compromise remain asymptomatic, which makes the imaging alone insufficient for making the diagnosis [1,2]. Additionally, the absence of radiologic findings does not exclude the diagnosis of TOS, as neurogenic TOS in particular may occur without any demonstrable abnormality on imaging [1]. 

This case series describes eight patients with TOS, highlighting the multimodality imaging findings across arterial, venous, and neurogenic subtypes and emphasising the importance of clinicoradiological correlation in diagnosis. 

## Materials and methods

Study design and population

This retrospective investigation was conducted at Sri Ramachandra Medical Centre, Chennai, Tamil Nadu, India. As this was a retrospective study involving anonymised patient data with no direct patient intervention, formal ethics committee approval and informed consent were not required per institutional policy. The study population comprised eight adult patients (aged 18 years or older) referred for diagnostic imaging based on clinical suspicion of TOS. Patients presented with a range of symptoms, including upper limb pain, paraesthesia, swelling, exertional venous engorgement, or signs of distal digital ischaemia. Cases were identified through a systematic query of the Radiology Information System (RIS) for TOS-related referrals between January 2020 and March 2026. Inclusion criteria required a clinical suspicion of TOS and the availability of diagnostic-quality imaging studies. For this study, diagnostic quality was defined as images free from significant motion artefact, adequate contrast opacification where applicable, and sufficient spatial resolution to allow confident assessment of the neurovascular structures at the thoracic outlet. Patients with non-diagnostic imaging or confirmed alternative diagnoses were excluded.

Imaging protocols

The imaging approach was tailored to each patient’s specific clinical presentation. Consequently, a multi-modality approach was utilised where necessary; however, the majority of patients underwent a single primary imaging study. The imaging approach was tailored to each patient's specific clinical presentation. Imaging modality selection was guided by the predominant clinical suspicion, and in cases where initial imaging yielded definitive findings, further investigation was not performed in order to avoid unnecessary radiation exposure and cost. A multimodality approach was reserved for cases where initial imaging was inconclusive or where overlapping subtypes were suspected. Only one patient in this series required both Doppler ultrasound and CT examinations for comprehensive evaluation.

Doppler Ultrasound

Examinations were performed using a high-frequency linear transducer (7-12 MHz, GE Logiq P10, GE HealthCare, Chicago, IL, USA) with the patient in an upright, seated position. The subclavian arteries and veins were initially evaluated in the neutral (adducted) position to establish baseline colour and spectral waveforms. This was followed by dynamic provocative manoeuvres, including arm abduction to 90° and 180° with contralateral head rotation and shoulder depression. Haemodynamically significant arterial compression was defined as a two-fold or greater increase in peak systolic velocity (PSV) during stress manoeuvres compared to the baseline neutral position, based on institutional Doppler practice and consensus agreement between the reviewing radiologists.

Computed Tomography (CT)

Contrast-enhanced CT was conducted on a 128-slice multidetector scanner (GE Revolution EVO, GE HealthCare, Chicago, IL, USA). For CT angiography (CTA), 80-100 mL of non-ionic iodinated contrast was administered via a power injector at a rate of 4 mL/s. Arterial-phase acquisition was triggered using bolus tracking in the ascending aorta, with coverage extending from the aortic arch through the distal subclavian arteries (slice thickness: 0.5-1.0 mm). For CT venography (CTV), images were acquired with a 45-60-second delay to ensure optimal opacification of the subclavian and axillary veins. When clinically indicated, dynamic CTA was performed with the symptomatic limb in an elevated position to assess for positional narrowing.

Magnetic Resonance Imaging (MRI)

Brachial plexus MRI was performed on a 3.0-tesla scanner (GE Signa Architect, GE HealthCare, Chicago, IL, USA) utilising a dedicated surface coil. The standardised protocol included axial T1-weighted spin-echo and axial/coronal T2-weighted fat-suppressed (short tau inversion recovery (STIR)) sequences, spanning from the C5 nerve root to the thoracic inlet. A slice thickness of 3-4 mm was maintained to facilitate precise visualisation of the interscalene triangle and costoclavicular space.

Image interpretation and analysis

All imaging studies were reviewed in consensus by two senior consultant radiologists, each possessing more than 15 years of experience. The reviewers systematically evaluated the images for anatomical variants (e.g., cervical ribs, anomalous first ribs, or fibrous bands) and soft-tissue abnormalities (e.g., muscular hypertrophy). For vascular cases, the degree of luminal narrowing, presence of intraluminal thrombosis, and development of collateral circulation were documented. Neurogenic involvement was assessed through secondary signs, such as the obliteration of regional fat planes or signal intensity changes within the brachial plexus. Each case was subsequently categorised as arterial, venous, or neurogenic TOS based on the correlation between predominant imaging findings and clinical symptoms.

## Results

A total of eight cases of TOS were analysed, including arterial, venous, and neurogenic subtypes (Table 1).

**Table 1:** Summary of demographic details, clinical presentation, imaging modality, and key findings in patients with thoracic outlet syndrome CTA, computed tomography angiography; TOS, thoracic outlet syndrome; PSV, peak systolic velocity; CTV, computed tomography venography; MRI, magnetic resonance imaging; M, male; F, female.

Case	Age/Sex	Symptoms	Imaging Modality	Key Findings	Final Subtype
1	29/F	Blackening of left third fingertip, burning pain, sensory loss	CTA	Focal narrowing of left subclavian artery at costoclavicular space, elongated C7 transverse processes	Arterial TOS
2	48/F	Right upper limb pain	Doppler ultrasound	PSV increase from 96 to 220 cm/s on arm elevation, preserved distal triphasic waveforms	Dynamic arterial TOS
3	28/M	Chronic upper limb pain, temperature intolerance	Doppler + CTA	Long-segment right subclavian artery occlusion (5.5 cm), anomalous first rib	Arterial TOS with chronic occlusion
4	42/M	Prominent left upper limb veins, mild pain, paraesthesia	CTV	Focal narrowing of left subclavian vein, no thrombosis, distal ectasia	Venous TOS (McCleery syndrome)
5	48/M	Exertional symptoms, asymmetric venous prominence	CTV	Subclavian vein compression by hypertrophied subclavius muscle, axillary collaterals	Venous TOS (muscular compression)
6	68/M	Bilateral limb swelling	CTV	Right subclavian vein narrowing, left cephalic vein compression, internal jugular thrombosis, AV fistula	Secondary venous TOS with multi-level obstruction
7	54/F	Left upper limb swelling, engorged neck veins	CTV	Left subclavian vein narrowing, pre/post-stenotic dilatation, extensive collaterals	Chronic venous TOS with collateralisation
8	16/M	Left upper limb weakness, supraclavicular swelling	MRI	Left cervical rib, thoracic outlet narrowing, vessel displacement, cervical lymphadenopathy	Neurogenic TOS

Case presentation 

*Case 1: Arterial TOS With Distal Ischaemia* 

**Figure 1:**
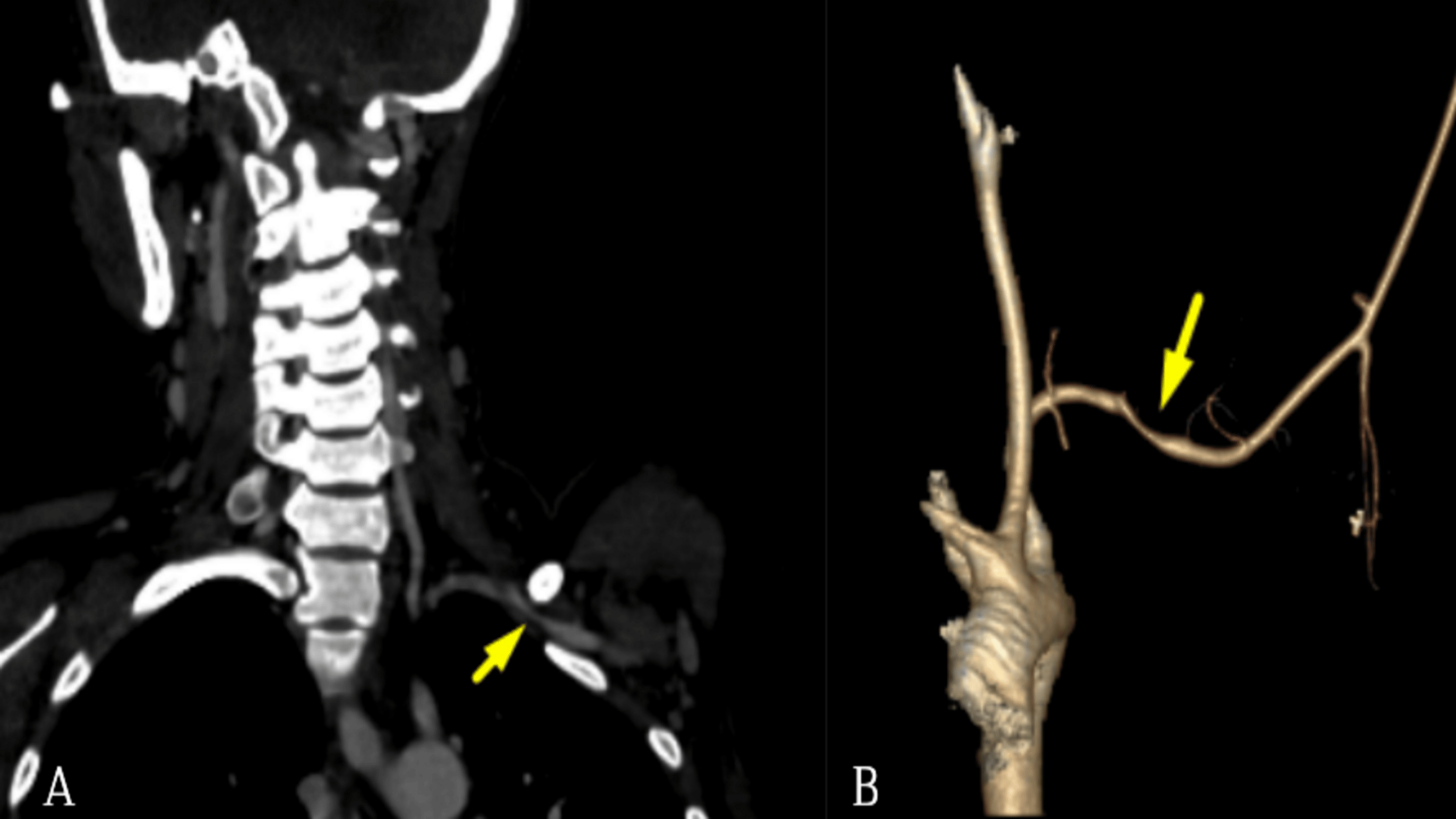
Subclavian artery compression on CT angiography (A) Coronal CT angiography demonstrating focal narrowing of the left subclavian artery (arrow) at the costoclavicular space. (B) Reformatted image demonstrating focal luminal narrowing of the left subclavian artery (arrow).

A 29-year-old female presented with sudden-onset blackening of the left third fingertip for a duration of two weeks. This clinical presentation was associated with burning pain and sensory loss involving the affected digit. Based on these symptoms, the initial clinical suspicion included Raynaud phenomenon as well as the possibility of vascular compression related to a cervical rib or other structural abnormality at the thoracic outlet. To further evaluate the vascular status and identify a possible site of compromise, CTA was performed. On coronal CTA imaging (Figure 1A), there was evidence of focal extrinsic compression involving the distal one-third of the left subclavian artery. This area of compression was localised to the costoclavicular space, which is a known anatomical site where vascular structures may be subjected to mechanical compression. Further assessment using reformatted images (Figure 1B) demonstrated that the involved arterial segment showed smooth luminal narrowing. The narrowed segment measured approximately 2.6 mm in diameter and extended over a length of approximately 2 cm. The smooth contour of the narrowing suggests an extrinsic compressive aetiology rather than an intrinsic luminal pathology. Despite the presence of this focal narrowing, distal arterial opacification was preserved, indicating that blood flow beyond the site of compression was still maintained. In addition, there was no evidence of intraluminal thrombosis or distal embolisation on the imaging study. These findings suggest that the stenosis is non-occlusive in nature. The degree of luminal narrowing was considered functionally significant in the context of the associated clinical presentation of distal digital ischaemia. In addition to the primary abnormality, an aberrant right subclavian artery arising from the distal aortic arch and coursing posterior to the oesophagus was identified. Elongation of bilateral C7 transverse processes, more pronounced on the left side, was also noted. However, these anatomical variations did not directly correspond to the site of compression observed in the left subclavian artery and therefore were not considered contributory in this case. When the clinical presentation is considered in conjunction with the imaging findings, the overall picture is consistent with arterial TOS, presenting in this instance with distal digital ischaemia.

*Case 2: Dynamic Arterial TOS* 

**Figure 2:**
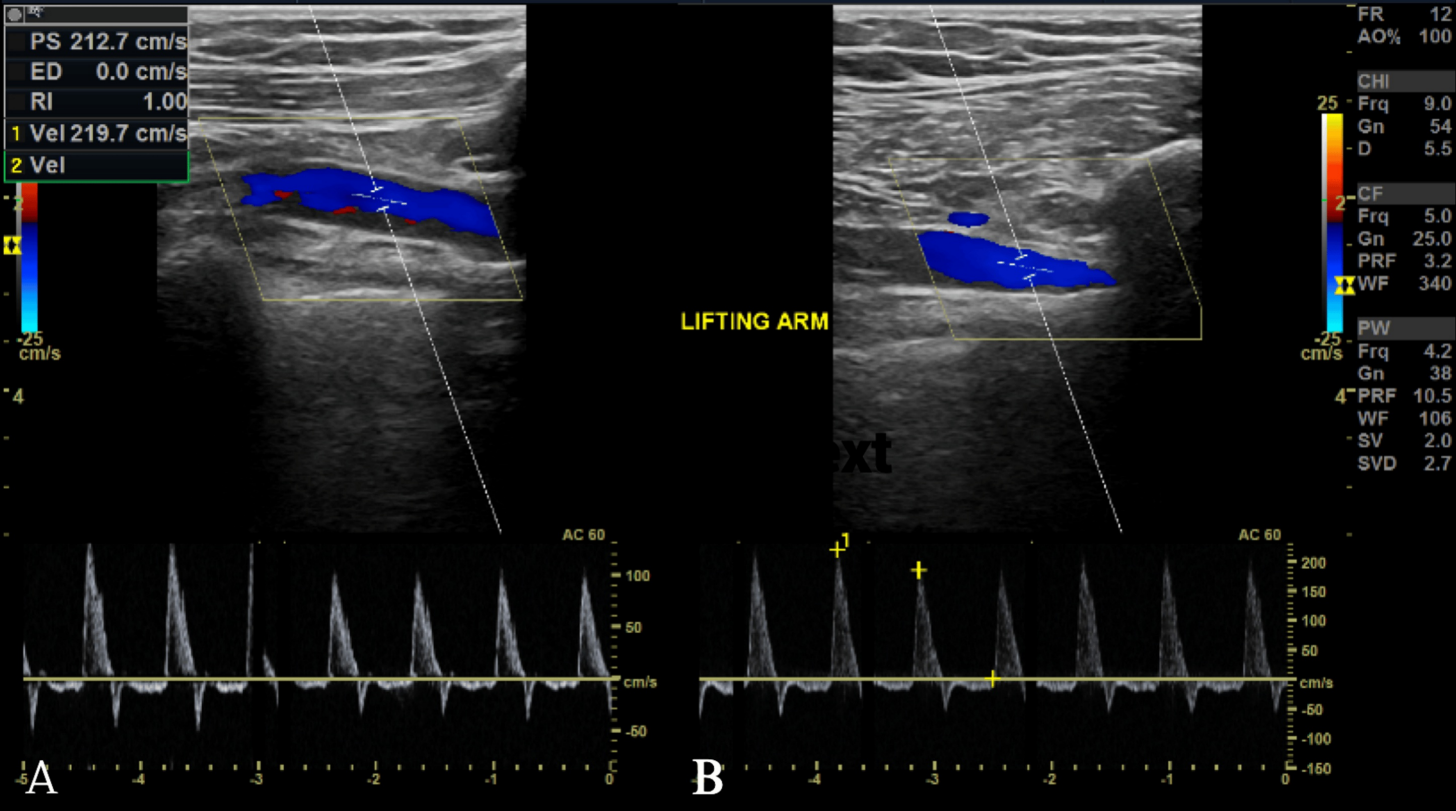
Dynamic arterial compression of the subclavian artery on Doppler ultrasound (A) Spectral Doppler waveform at rest demonstrating a normal triphasic pattern with a peak systolic velocity of 96 cm/s. (B) On arm elevation, there is a marked increase in the peak systolic velocity to 220 cm/s (>2-fold rise), consistent with dynamic arterial compression. Distal waveforms remain triphasic.

A 48-year-old female presented with right upper limb pain. In view of the presenting symptom, a vascular aetiology was considered, and Doppler evaluation of the right subclavian artery was performed to assess arterial flow characteristics both at rest and during positional change. At baseline, the Doppler study demonstrated a triphasic waveform in the right subclavian artery, with a PSV of 96 cm/s (Figure 2A). This finding is indicative of preserved arterial flow under resting conditions. However, on arm elevation, a significant change in flow dynamics was observed. Spectral Doppler imaging (Figure 2B) demonstrated an increase in PSV to 220 cm/s. This represents a greater than two-fold rise compared to the resting value, indicating the presence of haemodynamically significant stenosis that becomes apparent with positional change. Despite this marked increase in velocity at the level of compression, distal arterial waveforms remained triphasic. The patient reported reproduction of symptoms during arm elevation, supporting the clinical relevance of the dynamic compression. This observation is important, as it indicates that there is no fixed distal obstruction and that the haemodynamic alteration is positional rather than due to a persistent structural narrowing. When considered together, the increase in PSV with arm elevation and the preservation of distal triphasic waveforms support the diagnosis of dynamic arterial TOS. 

*Case 3: Arterial TOS With Chronic Occlusion* 

**Figure 3:**
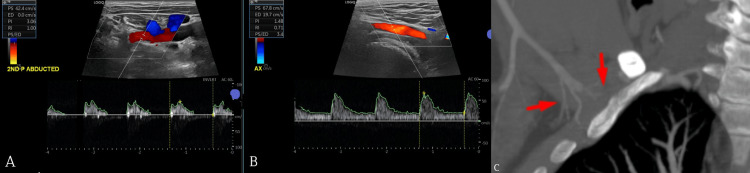
Chronic occlusion of the subclavian artery in arterial thoracic outlet syndrome (A) Doppler ultrasound demonstrating biphasic waveform in the distal subclavian artery. (B) Doppler ultrasound demonstrating monophasic waveform in the axillary artery. These findings are consistent with arterial occlusion with associated distal haemodynamic compromise. (C) Coronal CT angiography demonstrating long-segment occlusion of the right subclavian artery (arrows).

A 28-year-old male with a known aberrant right first rib presented with chronic upper limb pain and temperature intolerance. These clinical features raised concern for a long-standing vascular abnormality affecting arterial supply to the limb. Doppler evaluation was performed to assess arterial flow. The study demonstrated a biphasic waveform in the distal right subclavian artery (Figure 3A) and a monophasic waveform in the right axillary artery (Figure 3B). These waveform patterns indicate progressive dampening of arterial pulsatility, suggesting the presence of a proximal flow-limiting lesion. To further characterise the underlying pathology, CTA was performed. The CT coronal imaging (Figure 3C) demonstrated a long-segment occlusion of the right subclavian artery measuring approximately 5.5 cm in length. The occlusion was localised to the level of an anomalous first rib, which showed partial fusion with the second rib. This anatomical configuration results in narrowing of the costoclavicular space, providing a structural basis for chronic mechanical compression of the artery. The imaging findings correlate with the Doppler evidence of proximal flow limitation. Taken together, the clinical presentation, Doppler findings, and CT angiographic features are consistent with arterial TOS with chronic subclavian artery occlusion with associated haemodynamically significant proximal obstruction resulting in distal waveform dampening.

*Case 4: Venous TOS (McCleery Syndrome)* 

**Figure 4:**
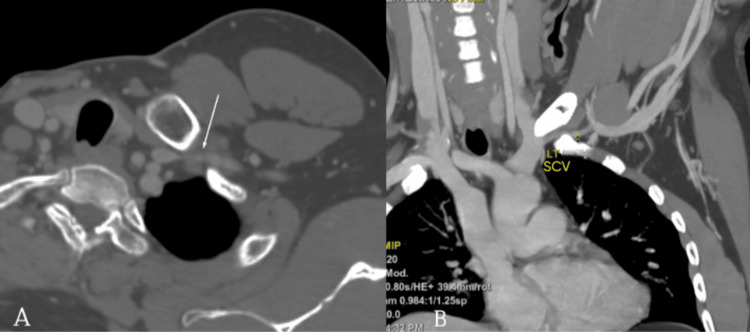
Venous compression without thrombosis (McCleery syndrome) (A) Axial CT venogram demonstrating focal narrowing of the left subclavian vein (arrow) at the costoclavicular space. (B) Coronal CT venogram confirming focal venous compression without evidence of intraluminal thrombosis, with mild distal venous ectasia. LT SCV, left subclavian vein.

A 42-year-old male presented with prominent left upper limb veins, which were noted to be accentuated during exertion. These symptoms were associated with mild pain and paraesthesia, suggesting an underlying disturbance in venous outflow. To evaluate the venous system, CTV was performed. Axial and coronal CT venogram images (Figure 4A, 4B) demonstrated severe focal narrowing of the left subclavian vein at the level of the costoclavicular space. Importantly, there was no evidence of intraluminal thrombus within the vein. However, distal to the site of narrowing, the venous segments showed mild ectasia along with fusiform dilatation. These findings reflect altered venous haemodynamics and are indicative of venous hypertension resulting from outflow obstruction. In the absence of thrombosis, the presence of focal extrinsic compression along with distal venous dilatation is characteristic of McCleery syndrome. Accordingly, the imaging findings in conjunction with the clinical presentation are consistent with venous TOS without thrombosis.

*Case 5: Venous TOS Due to Muscular Compression* 

**Figure 5:**
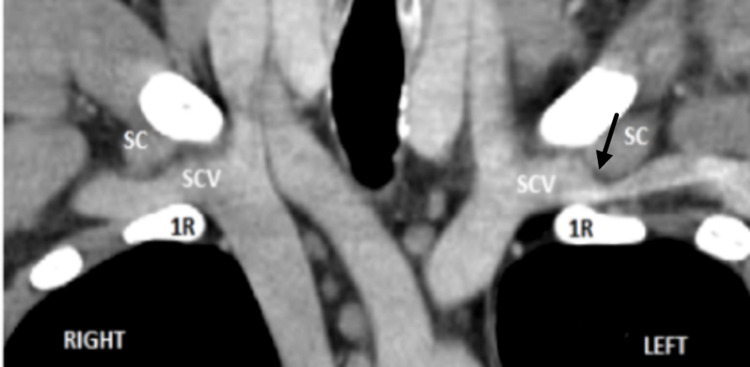
Muscular compression of the left subclavian vein by hypertrophied subclavius muscle Coronal CT venogram demonstrating compression of the left subclavian vein (arrow) between a hypertrophied subclavius muscle and the superior surface of the first rib. SC, subclavius muscle; SCV, subclavian vein.

A 48-year-old male presented with exertional symptoms along with asymmetry of the left upper limb venous prominence. These findings raised suspicion of a localised venous outflow obstruction. CT imaging was performed for further evaluation. Coronal CT venogram images (Figure 5) demonstrated compression of the left subclavian vein between a hypertrophied subclavius muscle and the superior surface of the first rib. This finding establishes the presence of extrinsic venous compression due to a muscular structure within the thoracic outlet. In association with this compression, venous collaterals were identified in the axilla and along the anterior chest wall, reflecting compensatory pathways for venous drainage. Mild contralateral indentation was also noted, although the dominant compression was observed on the left side. Taken together, these findings are consistent with venous TOS secondary to muscular compression.

*Case 6: Secondary Venous TOS With Multilevel Obstruction* 

**Figure 6:**
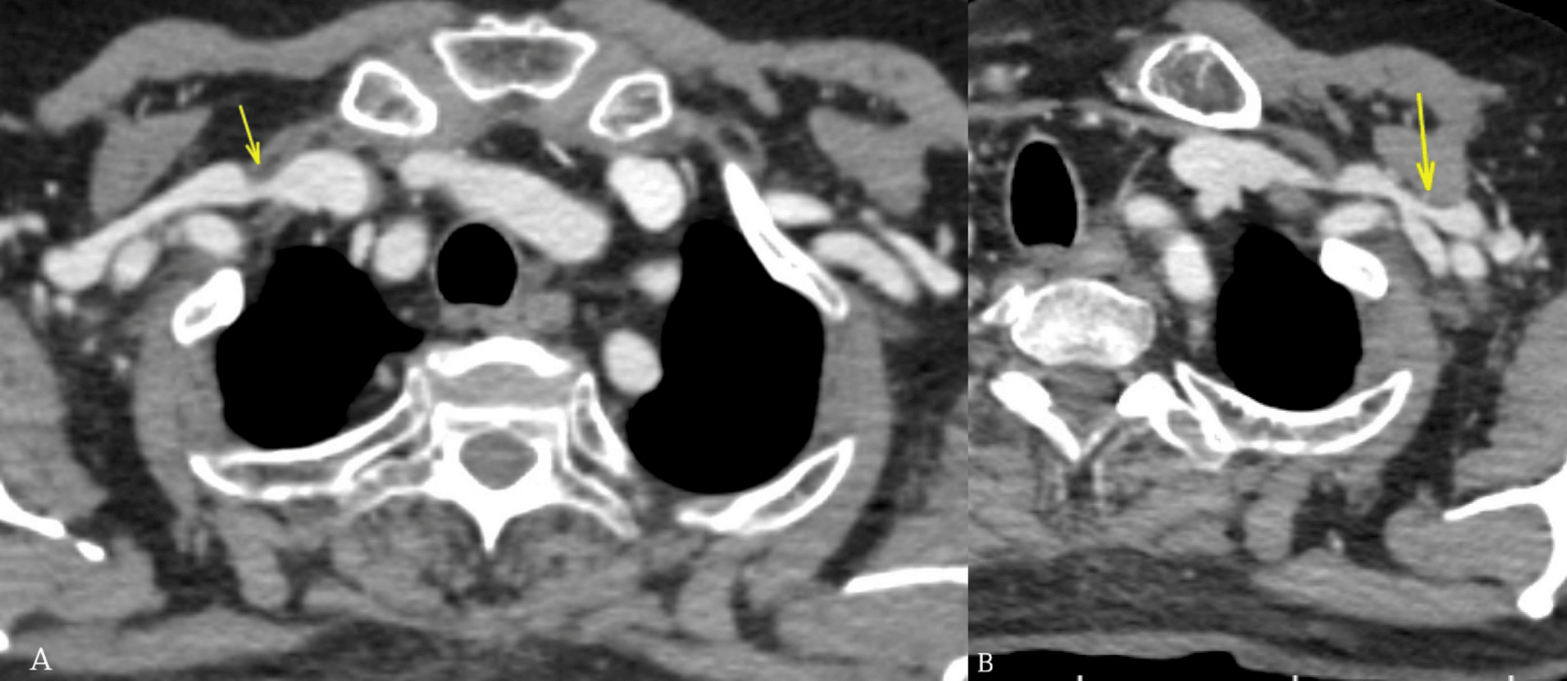
Multilevel venous obstruction in secondary venous thoracic outlet syndrome (A) Axial CT venogram demonstrating significant narrowing of the right subclavian vein at the costoclavicular space. (B) CT image showing subpectoral compression of the left cephalic vein by the pectoralis minor muscle, indicating multi-level venous obstruction.

A 68-year-old male with chronic kidney disease on haemodialysis presented with bilateral limb swelling. Given the clinical context, the possibility of central venous outflow obstruction was considered. CTV was performed to evaluate the venous system. Axial CT images (Figure 6A) demonstrated severe narrowing of the right subclavian vein at the level of the thoracic outlet within the costoclavicular space. In addition to this, further CT imaging (Figure 6B) demonstrated subpectoral compression of the left cephalic vein by the pectoralis minor muscle, indicating a second level of venous obstruction. The study also demonstrated chronic thrombosis of the internal jugular vein. An associated arteriovenous fistula with proximal venous narrowing was noted as well, which likely contributed to increased venous flow and venous hypertension, exacerbating central venous obstruction. These findings collectively indicate the presence of multilevel venous compromise, involving both the thoracic outlet and the subpectoral region. Accordingly, the imaging findings are consistent with secondary venous TOS with multilevel venous obstruction.

*Case 7: Chronic Venous TOS With Collateralisation* 

**Figure 7:**
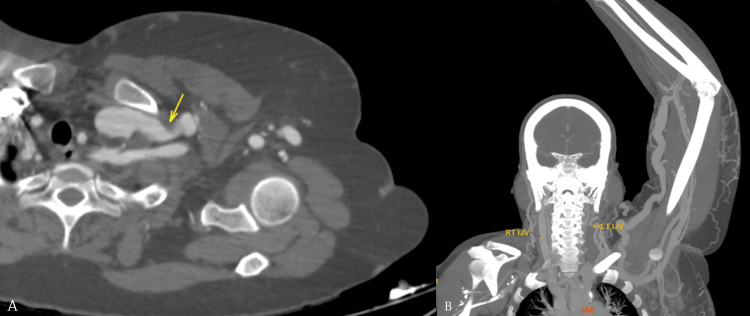
Chronic venous thoracic outlet syndrome with collateralisation (A) Axial CT venogram demonstrating focal narrowing of the left subclavian vein. (B) Coronal reformatted image showing marked pre- and post-stenotic dilatation with extensive collateral venous channels in the left upper limb and chest wall. RT IJV, right internal jugular vein; LT IJV, left internal jugular vein.

A 54-year-old female presented with left upper limb swelling and engorged neck veins, suggesting chronic venous outflow obstruction. CTV was performed for further evaluation. Imaging demonstrated focal narrowing of the left subclavian vein (Figure 7A). Coronal reformatted images (Figure 7B) further demonstrated marked pre-stenotic and post-stenotic dilatation along with tortuosity of the venous segments. In addition, extensive collateral venous channels were identified in the left upper limb, chest wall, and neck. These collateral pathways represent compensatory mechanisms that develop in response to long-standing venous obstruction. Associated chronic internal jugular vein narrowing was also noted. Taken together, these findings are consistent with chronic venous TOS with collateralisation.

*Case 8: Neurogenic TOS* 

A 16-year-old male presented with left upper limb weakness and supraclavicular swelling.

**Figure 8:**
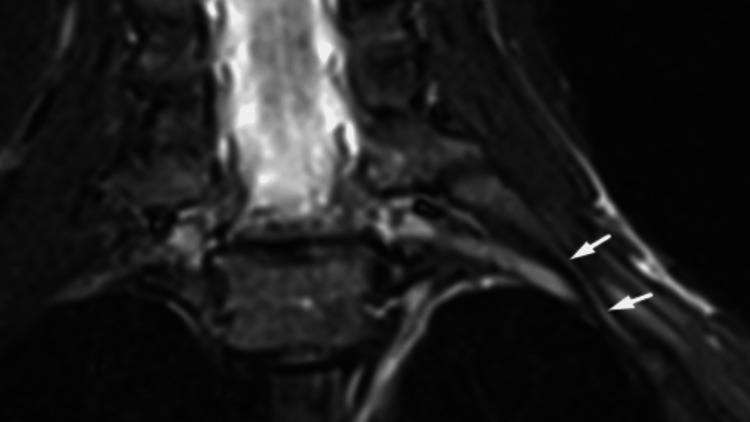
Cervical rib causing neurogenic thoracic outlet syndrome Coronal STIR MRI demonstrating a left cervical rib (arrows) causing narrowing of the thoracic outlet with displacement of adjacent subclavian vessels, suggestive of neurogenic compression. STIR, short tau inversion recovery; MRI, magnetic resonance imaging.

MRI of the thoracic outlet was performed using a coronal STIR sequence for evaluation of the neurovascular structures. The imaging (Figure 8) demonstrated a left cervical rib causing a reduction of the thoracic outlet space. This resulted in the displacement of the adjacent neurovascular structures, including the subclavian vessels. The reduction in available space within the thoracic outlet provides a structural basis for compression of the neural elements. In addition to this, there were also multiple prominent cervical lymph nodes at the site of supraclavicular swelling, which may contribute to supraclavicular swelling; however, they were not considered the primary cause of neurovascular compression. Their presence represents a potential confounding factor and highlights the need for careful differential evaluation. The direct brachial plexus signal changes were not demonstrated on MRI, nor was electrophysiologic correlation available; nonetheless, the structural abnormalities combined with the clinical presentation of left upper limb weakness are consistent with neurogenic TOS. 

## Discussion

TOS is a heterogeneous group of medical conditions described by compression of the neurovascular bundle through the thoracic outlet. Diagnostic uncertainty in TOS arises from the interactions between various anatomical structures, compression mechanisms, and the lack of agreement between imaging studies and clinical symptoms [1,2]. 

In this case series, arterial TOS showed more distinct and reproducible imaging characteristics compared with neurogenic TOS. Arterial TOS was commonly found in association with anatomic anomalies that caused either a fixed or dynamic narrowing of the vessel lumen, while venous TOS had a wider range, including simple focal compression, multi-level occlusion, and collateral development. In contrast, neurogenic TOS showed no consistent imaging signs, emphasising the clinical nature of this condition. 

Anatomical background and locations of the syndrome

Three major regions of the thoracic outlet include the interscalene triangle, the costoclavicular space, and the retropectoralis minor space [1,2]. Each area can act as a site for compression of the neurovascular bundle. Neurogenic TOS occurs when the brachial plexus becomes compressed at the interscalene triangle. While the subclavian artery passes through the same space, arterial involvement is not a defining feature of neurogenic TOS and should only be considered when specifically supported by clinical and imaging findings. The subclavian vein is more commonly compressed at the costoclavicular space [1]. Constituting factors contributing to the narrowing of the above structures are both congenital and acquired. Cervical ribs, fibrous bands, and other congenital causes, alongside trauma and repetitive upper-extremity motions, have been associated with developing TOS [1,4]. Nevertheless, the occurrence of anatomical variants in asymptomatic patients is essential for understanding one of the crucial limitations of the syndrome -- the presence of the abnormalities alone cannot be considered a criterion of the disease but should be taken into account only when related to the patient's symptoms [1,2]. 

Imaging findings depending on the subtypes of TOS

TOS can be divided into neurogenic, venous, and arterial subtypes [1,3]. Neurogenic TOS, being the most widespread among others, involves the brachial plexus and usually exhibits non-specific imaging manifestations [7]. MRI in some cases might demonstrate signs of neurogenic TOS in the form of perineural fat plane loss and changes in the appearance of the plexus [7]. Lack of specificity in imaging manifestations underlines that TOS, in most cases, is a clinical diagnosis with complementary imaging evaluation [5,7]. In turn, venous TOS, as a result of compression of the subclavian vein, is characterised by thrombus formation, focal stenosis, or development of collateral venous channels. While these imaging findings facilitate subtype identification, the diagnosis and management of venous TOS can remain complex, particularly in cases involving chronic thrombosis, extensive collateralisation, or dialysis-related central venous disease, as illustrated by several cases in this series.

On the contrary, in rare cases, TOS can manifest as arterial occlusion due to structural abnormalities like cervical ribs or anomalous first ribs [1,2]. Such a chronic process leads to injury of the vessel intima, thus provoking stenosis, dilation, aneurysmal change, thrombosis, and distal embolisation. All of the alterations are clearly identified on cross-sectional imaging [1]. 

Imaging role and limitations

Multimodal imaging is important in TOS diagnosis, and each imaging modality has specific strengths in this context [1]. Radiography can detect bony pathology, whereas ultrasound imaging provides a non-invasive assessment of blood vessel patency. CT and CTA provide high-resolution visualisation of bony pathology and vascular changes, particularly in cases of arterial diseases. MRI can assess soft tissue, including the brachial plexus, and evaluate indirect evidence of nerve compression [1,7]. Nonetheless, imaging results have to be carefully assessed because of the limited specificity of some radiologic features, since some changes (positional vascular narrowing and anatomical variants) can occur in asymptomatic people as well [1,2]. Therefore, imaging techniques can only complement the clinical assessment [1,2,8]. 

Differential diagnosis

Other conditions, such as apical lung masses, peripheral nerve sheath tumours, large vessel vasculitis, and atherosclerosis, can produce similar symptoms [1]. Detailed examination using imaging tools is crucial in order to differentiate TOS from those conditions. 

Clinical consequences

Overall, the results of the case series illustrate the relevance of clinical integration in TOS diagnosis. Although imaging plays a major part in detecting anatomic abnormalities and vascular changes, it provides more diagnostic information if combined with clinical signs. Key considerations include the greater clinical significance of demonstrable vascular abnormalities compared to isolated anatomical compression, necessitating careful evaluation. Imaging primarily serves to support and refine clinical suspicion rather than establish the diagnosis independently.

Limitations include small sample size, retrospective design, non-uniform imaging protocols, lack of surgical confirmation, limited follow-up, and absence of electrophysiological validation.

## Conclusions

This case series illustrates the broad imaging spectrum of TOS across its arterial, venous, and neurogenic subtypes. The cases demonstrate that TOS may arise from diverse mechanisms, including dynamic vascular compression, fixed anatomical narrowing, chronic occlusive disease, muscular impingement, and multilevel venous obstruction, each with distinct imaging appearances. These findings reinforce that anatomical variations alone, such as cervical ribs or muscular hypertrophy, are insufficient to establish a diagnosis, as they may also be present in asymptomatic individuals. Accurate diagnosis, therefore, requires careful clinicoradiological correlation, particularly in neurogenic TOS, where imaging findings may be subtle or absent. However, the conclusions drawn from this series are limited by the small sample size, retrospective design, non-uniform imaging protocols, and absence of surgical or electrophysiological confirmation. Further validation in larger, prospective, standardised studies is required to better define the diagnostic role of multimodality imaging in TOS.
